# Correlates of substitution rate variation in mammalian protein-coding sequences

**DOI:** 10.1186/1471-2148-8-53

**Published:** 2008-02-19

**Authors:** John J Welch, Olaf RP Bininda-Emonds, Lindell Bromham

**Affiliations:** 1 Institute of Evolutionary Biology; School of Biological Sciences; University of Edinburgh, West Mains Rd., Edinburgh EH9 3JT, UK; 2 Centre for the Study of Evolution; School of Life Sciences; University of Sussex, Falmer, Brighton BN1 9QG, UK; 3 Institut für Spezielle Zoologie und Evolutionsbiologie mit Phyletischem Museum; Friedrich-Schiller-Universität Jena, Erbertstrasse 1, 07743 Jena, Germany; 4 Centre for Macroevolution and Macroecology; School of Botany and Zoology; Australian National University, Canberra, A.C.T. 0200, Australia

## Abstract

**Background:**

Rates of molecular evolution in different lineages can vary widely, and some of this variation might be predictable from aspects of species' biology. Investigating such predictable rate variation can help us to understand the causes of molecular evolution, and could also help to improve molecular dating methods. Here we present a comprehensive study of the life history correlates of substitution rate variation across the mammals, comparing results for mitochondrial and nuclear loci, and for synonymous and non-synonymous sites. We use phylogenetic comparative methods, refined to take into account the special nature of substitution rate data. Particular attention is paid to the widespread correlations between the components of mammalian life history, which can complicate the interpretation of results.

**Results:**

We find that mitochondrial synonymous substitution rates, estimated from the 9 longest mitochondrial genes, show strong negative correlations with body mass and with maximum recorded lifespan. But lifespan is the sole variable to remain after multiple regression and model simplification. Nuclear synonymous substitution rates, estimated from 6 genes, show strong negative correlations with body mass and generation time, and a strong positive correlation with fecundity. In contrast to the mitochondrial results, the same trends are evident in rates of nonsynonymous substitution.

**Conclusion:**

A substantial proportion of variation in mammalian substitution rates can be explained by aspects of their life history, implying that molecular and life history evolution are closely interlinked in this group. The strength and consistency of the nuclear body mass effect suggests that molecular dating studies may have been systematically misled, but also that methods could be improved by incorporating the finding as *a priori *information. Mitochondrial synonymous rates also show the body mass effect, but for apparently quite different reasons, and the strength of the relationship with maximum lifespan provides support for the hypothesis that mtDNA damage is causally linked to aging.

## Background

There is now a great deal of evidence that rates of DNA substitution can vary widely between closely related lineages [1-3], and while some of this variation is erratic and locus-specific, trends also apply consistently across many loci [2,4-7]. There is also increasing evidence, particularly in vertebrates, that some of this lineage-specific rate variation may be predictable from aspects of a species' biology [8-17]. Uncovering this predictable rate variation is an important part of understanding the causes of molecular evolution [18]. It may also bring practical benefits, because variation in the rate of substitution complicates the production of dated molecular phylogenies, which are increasingly relied upon in diverse areas of biology. If reliable correlates of rate variation can be identified, this information could be exploited to improve molecular dating methods [15,16,19,20].

The present study investigates the causes and correlates of lineage-specific rate variation in mammalian protein coding sequences. Mammals were chosen because of the unrivalled availability of relevant data: mitochondrial and nuclear DNA sequences, life-history records and comprehensive phylogenetic information [21,22]. In addition, the timings of ordinal and higher-level radiations of the mammals have been controversial, and systematic change in substitution rates has been proposed as an explanation of disagreements between molecular and palaeontological dating approaches [23-27].

Many previous studies have tested for correlates of mammalian rate variation [5,6,14,15,17,28-32], but no clear consensus has emerged. One problem is that negative findings have been difficult to interpret because of issues surrounding statistical power in the comparative study of substitution rates. In some cases, sample size was inflated artificially by a failure to correct for phylogenetic non-independence. This means that single instances of evolutionary change can be counted multiple times, and this can increase both false positive and false negative error rates [33,34]. Even studies that did take shared ancestry into account, e.g. [14], did not always use appropriate statistical methods or diagnostic tests of the parametric assumptions, and this too can yield misleading results [35,36]. The question of statistical power is particularly vexed in the study of substitution rates, because rates cannot be measured directly, but must be inferred from DNA sequence data. For short sequences, or short periods of divergence, a small number of substitutions can make a large difference to the rate inferred, making the estimates very noisy [16,37]. Accordingly, there is often a trade-off between the number of data points in an analysis, and the accuracy with which each data point estimates a change in rate. In the present study, we address these problems by using large multi-gene alignments, and the method of phylogenetically independent contrasts [33,34], combined with new procedures for establishing a minimum comparison depth [37].

Another difficulty, particularly acute for mammals, stems from the widespread correlations between all aspects of their life histories and wider biology; these include not just strong and ubiquitous allometries, but also strong correlations between traits after correction for body size [22,38-40]. These colinearities make regression analyses particularly difficult to interpret, because a significant result can always be plausibly attributed to some absent variable covarying with the predictor. For example, many comparative studies of mammalian aging have been criticised for failing to take into account the covariation of lifespan with body mass [13,41,42]. Here, the problem of covarying life history traits is mitigated by including, in a single multiple regression analysis, most of the correlates of vertebrate rate variation that have been identified or hypothesised in the literature. Specifically, the variables included are body mass [13,14,20,28], which has often been used as a proxy for basal metabolic rate [11,15,17,43-45], organismal generation time [8,9,14,28], fecundity [10,46], and maximum recorded lifespan [13,32,41]. These variables are defined in detail below, and an assessment of the various causal hypotheses with which they have been associated follows in the discussion.

## Results

### Mitochondrial rates

**Figure 1:**
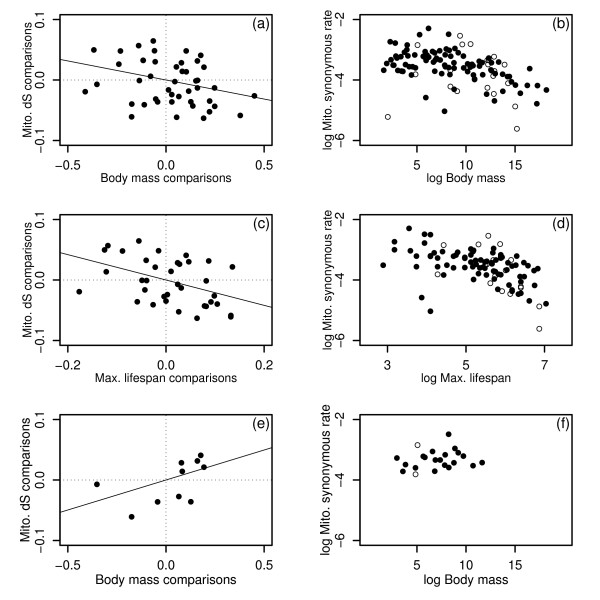
**Mitochondrial results**. Plots of mitochondrial synonymous substitution rate (expected substitutions per site per million years), against body mass in grammes (a-b), and maximum recorded lifespan in months (c-d). Substitution rates were estimated from a concatenated alignment of nine loci. Shown are phylogenetically-independent comparisons, (a) and (c), with the best-fit regression line forced through the origin, and the raw cross-species values (b) and (d), with lineages excluded from the independent comparisons analysis shown as empty circles. For the independent comparisons, trait measurements were log transformed, and contrasts standardised with their expected standard deviation [see Additional file 2]. (e) and (f) show body mass results for the Euarchontoglires, a subset of the data in (a) and (b).

The single variable regressions, summarised in Table 1, show that of the four variables tested, body mass and maximum lifespan are individually significant predictors of mitochondrial synonymous substitution rates: Species with greater mass, or longer lifespan tend to have slower rates of synonymous substitution. The significant regressions are plotted in Figure 1a–d, together with their associated raw cross-species plots, which show closely similar trends. A multiple regression analysis, including pairs with measurements for all four predictor traits, shows that only maximum lifespan remains significant (Table 2). Furthermore, model simplification shows that lifespan alone explains almost as much variation as does the four-predictor model (Table 2). Table 3 contains results for the larger subset of points for which lifespan and body mass measurements were available (i.e., including those pairs lacking fecundity or generation time data). Again, maximum lifespan was found to be the sole significant predictor. Diagnostic tests identified the sperm whale pair, *Kogia breviceps*-*Physeter catodon*, as a weak outlier, and excluding this pair increases the *r*^*2 *^to levels matching those in Table 2. (It is possible that the outlying nature of this point reflects the recognised difficulties obtaining accurate measurements of maximum lifespan for cetaceans: [47].)

**Table 1:** Single predictor regressions

Rate	Trait	*n*	slope	*r*^*2*^	*p*-value
Mito. *dS*	Body mass	45	-0.06	0.105	0.028*
	Max. lifespan	36	-0.21	0.195	0.0062*
	Generation time	28	-0.15	0.057	0.21
	Fecundity	28	0.40	0.011	0.60
Mito. *dN*	Body mass	45	0.01	0.001	0.88
	Max. lifespan	36	0.11	0.030	0.31
	Generation time	28	0.03	0.001	0.87
	Fecundity	28	1.66	0.076	0.15

Nucl. *dS*	Body mass	16	-0.09	0.384	0.0079*
	Max. lifespan	16	0.03	0.007	0.75
		14†	-0.21	0.273	0.046*
	Generation time	13	-0.18	0.330	0.032*
	Fecundity	12	4.06	0.535	0.0045**
Nucl. *dN*	Body mass	16	-0.13	0.471	0.0023**
	Max. lifespan	16	-0.04	0.010	0.70
		14†	-0.24	0.146	0.13
	Generation time	13	-0.16	0.149	0.17
	Fecundity	12	4.24	0.488	0.0080*

Note:- * *p *< 0.05, ** *p *< 0.005. † Pairs with outlying lifespan values removed.

**Table 2:** Four-variable multiple regressions

Rate	Model			Coefficients: slope (*p*-value)			
		
	*n*	*r*^*2*^	*p*-value	Body mass	Max. lifespan	Gen. time	Fecundity
Mito. *dS*	22	0.352	0.084	-0.014 (0.741)	-0.246 (0.019)*	-0.019 (0.893)	0.103 (0.916)
		0.350	0.014*	-0.020 (0.561)	-0.246 (0.014)*		
		0.338	0.004**		-0.268 (0.004)**		
	14†	0.698	0.011*	-0.098 (0.113)	-0.244 (0.029)*	0.124 (0.527)	-0.685 (0.459)
		0.673	0.001**	-0.059 (0.107)	-0.238 (0.022)*		
		0.590	0.001**		-0.334 (0.001)**		
Mito. *dN*	22	0.112	0.670	0.009 (0.907)	0.156 (0.395)	0.145 (0.585)	2.263 (0.219)

Nucl. *dS*	11	0.822	0.009*	-0.038 (0.229)	0.240 (0.093)	-0.250 (0.060)	2.138 (0.099)
		0.777	0.005*		0.209 (0.138)	-0.281 (0.039)*	2.537 (0.055)
		0.702	0.004**			-0.130 (0.112)	2.390 (0.084)
Nucl. *dN*	11	0.681	0.062	-0.095 (0.078)	0.017 (0.934)	0.118 (0.531)	3.069 (0.134)

Note:- * *p *< 0.05; ** *p *< 0.005. † Euarchontoglires removed.

**Table 3:** Body size and maximum lifespan regressions

Data			Model		Coefficients: slope (*p*-value)	
		
Rate	pairs excluded	*n*	*r*^*2*^	*p*-value	Body mass	Max. lifespan
Mito. *dS*	None	36	0.219	0.015*	-0.033 (0.317)	-0.165 (0.049)*
	*Kogia *– *Physeter*	35	0.309	0.0022**	-0.046 (0.152)	-0.213 (0.013)*
	*Euarchontoglires*	27	0.359	0.0038**	-0.071 (0.074)	-0.127 (0.190)
	*Euarchontoglires *+ *Kogia *– *Physeter*	26	0.512	0.00018**	-0.087 (0.017)*	-0.184 (0.043)*

Nucl. *dS*	None	16	0.492	0.0088*	-0.111 (0.003)**	0.112 (0.108)
	Lifespan outliers	14	0.473	0.022*	-0.081 (0.054)	-0.037 (0.764)
			0.468	0.0049*	-0.089 (0.005)**	
Nucl. *dN*	None	16	0.496	0.0083*	-0.137 (0.003)**	0.066 (0.426)
	Lifespan outliers	14	0.486	0.018*	-0.143 (0.019)*	0.077 (0.651)
			0.477	0.0044**	-0.126 (0.004)**	

Note:- * *p *< 0.05, ** *p *< 0.005.

To check for consistency across the major superordinal groups, we carried out separate analyses for the three clades with sufficient comparisons, namely Metatheria (marsupials), Laurasiatheria (Cetartiodactyla, Carnivora, Perissodactyla, Chiroptera, and some former Insectivora) and Euarchontoglires (Rodentia, Lagomorpha, Scandentia, Dermoptera and Primates) [see Additional file 1]. For both Metatheria and Laurasiatheria, results for all traits are closely consistent with each other, and with the complete data set (for example, for body mass we have Metatheria: *n *= 11, slope = -0.06, *r*^*2*^*= *0.22; Laurasiatheria: *n *= 21, slope = -0.11, *r*^*2*^*= *0.24). For the Euarchontoglires, by contrast, lifespan, fecundity and generation time had slopes close to zero, and no visible trend in the raw cross-species plots. Furthermore, in this group synonymous rates actually appear to increase with body mass (Figure 1e–f), albeit with a non-significant regression (Euarchontoglires: *n *= 10, slope = 0.10, *r*^*2*^*= *0.25, *p *= 0.12). Accordingly, we repeated the full analyses with the Euarchontoglires excluded. For the two-variable regression (Table 3), results were not robust, with neither predictor reaching significance for the 27 remaining pairs, but both reaching significance when the outlying sperm whale pair was excluded (Table 3). However, for the subset of pairs with all variables measured, maximum lifespan was again identified as the sole significant predictor (Table 2). Excluding Euarchontoglires also led to *r*^*2*^ values that were much higher in both cases (Tables 2, 3).

In stark contrast to results for synonymous substitutions, mitochondrial nonsynonymous rates show no trend with any of the predictor variables (Tables 1 and 2). This applied equally to the independent comparisons, and to the raw cross-species plots (not shown).

### Nuclear rates

**Figure 2:**
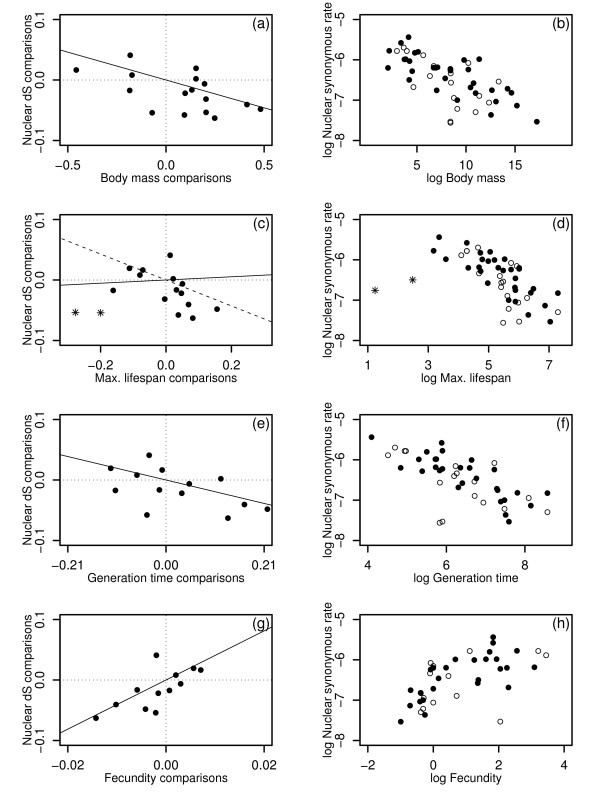
**Nuclear results**. Plots of nuclear synonymous substitution rate against body mass (a-b), maximum recorded lifespan (c-d), generation time (e-f), and fecundity (g-h). Substitution rates were estimated from a concatenated alignment of six loci. Two species with outlying lifespan values are shown as asterisks in (c) and (d), and (c) includes the regression lines for both the complete set of points (solid line), and with these two outlying values excluded (dashed line). All other details are as for Figure 1.

Plots for the nuclear synonymous data set are shown in Figure 2. Table 1 shows that body mass, generation time and fecundity are all significant predictors of nuclear synonymous rates. Lifespan, by contrast, shows no trend, but this is attributable to two clear outliers, appearing as such in both the diagnostic tests of the regressions (Fig. 2c) and in the raw cross-species scatter-plots (Fig. 2d). (The two small values are for *Cynocephalus variegatus*, the malayan flying lemur, and *Amblysomus hottentotus*, the hottentot golden mole). Removing these two outlying points makes maximum lifespan, too, a significant predictor of synonymous rates (Table 1). Of course, these outliers could represent true biological variation, but problems with maximum recorded lifespan as a statistic make measurement error particularly likely in this case [13,22,47,48]. (For example, recorded lifespan can only increase with new observations, and so is strongly dependent on the number of animals sampled; a species' suitability for captivity will also have an important influence.) If outliers are excluded, then for each of the predictors, *r*^*2*^ values are much higher than for the mitochondrial data set, even when corrections for reduced sample size are made. Furthermore, and again in contrast to the mitochondrial results, all effects apply consistently to Laurasiatheria and Euarchontoglires (the nuclear data set contains no Metatheria).

The multiple regression results for the nuclear data (Table 2) are unfortunately difficult to interpret. Model simplification leads to a model with generation time and fecundity remaining. But neither coefficient is individually significant, and model fit is poor in several respects (for example, the Shapiro-Wilks test shows significant departures from normality). More telling are the two-variable regressions with body mass and lifespan (Table 3). For nuclear rates, body mass is found to be the superior predictor, whether or not the outlying lifespan values are excluded, and, in either case, lifespan drops out in model simplification. This is the opposite result to that obtained for the mitochondrial data set (Table 3).

Finally, and again in direct contrast to the mitochondrial results, for the nuclear data set the same trends are observed for both synonymous and non-synonymous rates (Tables 1, 2, 3). While the non-synonymous data are noisier, with generation time and longevity effects not reaching individual significance, the slopes of the regressions are very similar (Table 1), and body mass is again favoured over maximum lifespan in multiple regression (Table 3).

## Discussion

It is often possible to attribute an apparent change in the rate of substitution to some other factor, such as misspecification of the substitution model, inaccurate divergence dates, changes in base composition, or a transient and locus-specific burst of adaptive changes. But the identification of significant predictors of rates, within the framework of phylogenetically independent comparisons (Table 1; Figures 1, 2), is difficult to reconcile with any of these explanations, and argues strongly that changes of substitution rate are frequent and substantial within the mammals. Conversely, the various sources of error inherent in the estimation of rates makes it probable that the *r*^*2*^ values estimated here – high as they are – are systematic underestimates of the true relationship between rate and trait. (Specifically, rate differs from traits such as body mass, in that it must be estimated from the number of substitutions accrued stochastically over a period of time, which must in turn be estimated from comparison of sequence data, and all of these factors can obscure the true relationship between trait and rate [37].)

### Implications for molecular dating

From the perspective of molecular phylogenetics, the strength and consistency of the body size effect across our nuclear data set is of particular interest: If such an effect applies, concerted changes in the average body size of a clade over time can create systematic biases in the molecular date estimates for that clade, even if variable-rate dating methods are used [16,19,49]. The same biases can apply in the absence of concerted body size change, if larger or smaller taxa are over-represented in the data sampled. For these reasons, if there was a concerted increase in mammalian size between the late Cretaceous and early Tertiary, or if larger mammals have been disproportionately sampled, then molecular date estimates of the ordinal level radiation of mammals could have been systematically misled [16,23-26]. While the widespread rate variation presents a problem for molecular dating, the identification of robust predictors of rate gives grounds for optimism. In particular, body size is both widely sampled for extant species, and can be estimated for extinct or ancestral taxa from their fossil record. Consequently, this information could be used to improve future molecular dating methods, allowing the development of "corrected molecular clocks", or empirically informed priors for Bayesian approaches [15,19,20].

### Mitochondrial rate variation, and mitochondrial theories of aging

For the mitochondrial data set, maximum lifespan was found to be the strongest predictor of synonymous rate (Table 1), and was the sole significant predictor in multiple regressions (Tables 2, 3). This implies that the mitochondrial body size effect, e.g., [14] can be attributed solely to the correlation of mammalian body mass and lifespan [13,39-41]. In contrast, for the nuclear data set, maximum lifespan dropped out of the model in favour of body mass – even when outlying lifespan values were removed (Tables 1, 2, 3). Together, these findings support the longstanding, though still controversial, theory that mitochondrial DNA damage is causally linked to aging [17,50-54]. The possibility that this link is causal is strengthened, ironically, by the many problems with maximum recorded lifespan as a statistic [13,47,48]. It has been shown many times that maximum lifespan is a poor proxy for typical longevity in the wild, with the two quantities differing substantially and unpredictably in many cases [55,56]. As such, maximum recorded lifespan relates only weakly to the realised life histories on which selection can act [39,40,57].

A link between mtDNA damage and mammalian aging also has some direct experimental support. Premature aging has been reported in mice expressing defective mitochondrial DNA polymerase [58-60], and an extension of youthful lifespan shown to result from the over-expression of mitochondrially-targeted catalase, but not of nuclear-targeted catalase [61]. However, there are also reports that mitochondrial point mutations have no effect on mouse lifespan [62,63]. These latter results might be consistent with recent modifications of the mitochondrial theory of aging, implicating mtDNA deletions rather than point mutations [51,52,54], and consistent with our results if the two types of DNA damage are highly correlated in nature.

Although the precise mechanisms linking mtDNA damage to senescence are much disputed, most theories implicate Reactive Oxygen Species (ROS): Mitochondria are the major site of ROS production, and mtDNA a major site of oxidative damage [50-52]. ROS production is an inevitable by-product of aerobic metabolism, and this raises the possibility that the true determinant of substitution rates is basal metabolic rate (BMR) [11,15,43,64]. BMR was not included in the present study, but there are several reasons to believe that its inclusion would not have altered our conclusions. First, the correlation of BMR with body mass is particularly strong in mammals [44,65], making it very unlikely that lifespan would be identified as a better predictor than body size if BMR were the true causal factor. Second, the mechanistic basis of the metabolic rate hypothesis is doubtful, both because the basal rate is a poor measure of total energy metabolism [65], and because mammalian metabolism can be decoupled from ROS production by various means [45,65,66]. Nuclear DNA, where the body mass effect is strongest (Tables 1 and 3), also appears to be protected from mitochondrially-generated ROS [67]. Third, there is some evidence that mtDNA damage can accelerate aging without increasing oxidative stress [59,60]. Finally, previous analyses of substitution rates have not identified BMR as a significant predictor when body size was also included in the model [12,14,45].

Particularly revealing case studies come from species whose ecologies have led to departures from the mammalian norm. For example, the naked mole rat, particularly long-lived and metabolically slow for its size, shows peculiarly low levels of anti-oxidative defences, and correspondingly high levels of oxidative damage in its nuclear DNA [68], but this might be attributable, paradoxically, to reduced levels of ROS production in the mitochondria, implying reduced levels mtDNA damage [68,69]. Just such a situation appears to explain the greater longevity of birds compared to mammals [52]. It would also be of great interest to compare patterns of rate variation in Chiroptera, a group not well represented in the present study [17,47,48,57,70].

A final puzzling aspect of the mitochondrial results is the anomalous patterns shown by the Euarchontoglires – the group that contains both rodents and primates, and so all of the most highly studied mammalian models. For this superordinal group, synonymous rate appears to increase with body mass (Fig. 1e–f), and shows no trend with lifespan. One possible explanation is that these results reflect the confounding influence of weak purifying selection. Theory has shown that, if certain assumptions hold, we can expect a negative correlation between the mutation rate per year, and the fixation probability of weakly deleterious mutants [18,28,71]. For example, if species with higher mutation rates also have larger populations, then the increased efficacy of purifying selection could lead to a reduction in the rate of substitution due to genetic drift. It is possible that weak purifying selection acts on mitochondrial synonymous sites in mammals [72], and there is also some evidence that the positive relationship between mutation rate and population size does hold [73]. This effect is likely to be dampened by the complete linkage of mitochondrial DNA, which weakens the dependence of substitution rate on population size [71,74], but typical mammalian populations may be small enough for drift-based effects to remain important [75]. Furthermore, when linkage is tight, a negative correlation between mutation rate and the fixation probability could still hold, if populations with higher mutation rates per year, also have higher rates of adaptive substitution per generation [71].

The hypothetical explanation given above remains incomplete, however, because it remains unclear why effects counteracting the variation in mutation rates should be so much stronger in the Euarchontoglires than in the other major mammalian groups.

### Nuclear rate variation and mammalian life history

Compared to the mitochondrial data, our nuclear data set is smaller in every respect, and particularly in terms of the fraction of the genome sampled. However, for the loci studied, the influence of life history on substitution rate appears to be even more pervasive.

For example, in common with previous studies, no successful predictor was found for mitochondrial non-synonymous changes [5,14,30], but our nuclear results were similar for both classes of site (Tables 1, 2, 3). The nuclear results contrast with some previous empirical studies [14,29], and also contradict theoretical predictions, mentioned above, that the evolution of selected sites should be relatively immune to variation in life history [71]. We cannot know whether our nuclear data set is representative, in this respect, of nuclear loci in general, but examination of the *dN*/*dS *ratios yields no evidence that levels of selective constraint are anomalous (values for the 32 lineages in the main data set range from 0.046 to 0.200 [see Additional file 1], which can be compared to results from complete genomes of primates and rodents [76]). Furthermore, the theoretical predictions rely on assumptions that are unlikely to hold at all loci (e.g., the influence of population size on levels of selective constraint may be slight for highly leptokurtic distributions of selective effects [18]). Our results are also consistent with observations that a substantial component of nuclear nonsynonymous rate variation in mammals is lineage-specific [2], and with the strong correlation between nuclear *dS *and *dN *[7] (for contrasting patterns in mitochondrial sequences, see [2,5,72]).

Results for our nuclear loci also contrast with the mitochondrial patterns in terms of the relative success the individual predictors. A strong effect of lifespan on nuclear rates can be discounted, but no single other factor was unambiguously favoured (Table 2). Nevertheless, the contrast between the two sets of results allows us to draw some tentative conclusions about various causal hypotheses.

For example, the argument for body mass as a true causal factor stems from evidence that larger bodied mammals suffer increased risk of cancer [13,42], and that this might select for increased DNA repair [13,41,77]. But this hypothesis is difficult to reconcile with the comparative weakness of the body size effect in mtDNA, where links between mutation and carcinogenesis are well established [62,77]. In contrast, the success of generation time as a predictor of nuclear, but not mitochondrial rates, might reflect differences in the biology of the two genomes. In particular, germline mosaicism [78], where a large fraction of nuclear mutations appear in just one or two meiotic divisions, is certain to strengthen the correlation between generation time and mutation rate per year; while the replication of mtDNA is potentially decoupled from cell division [72], weakening such a dependency.

A more perplexing result is the apparent increase in nuclear rates with fecundity. Non-significant for mitochondrial rates, this predictor has an *r*^*2*^ of around 50% for both classes of nuclear site (Table 1). It has been hypothesised that increases in fecundity might entail an increased mutation rate [46], or reflect increased variance in offspring viability, and so lower effective population size [79]. But given the limited variation in mammalian fecundity, it is unlikely that either of these explanations could explain our results [22,80]. A third intriguing possibility views germline mutation rate as an integral part of life history strategy [22,39,40], suggesting that species with fewer offspring should invest more heavily in each, implying a selective pressure to lower germline mutation rates [10].

## Conclusion

We have presented a comprehensive study of substitution rate variation in protein-coding sequences across the mammals, and shown that for mitochondrial synonymous sites, and for both synonymous and nonsynonymous sites in 6 nuclear loci, a substantial fraction of the between-lineage rate variation can be explained by aspects of life history.

The results imply that molecular dating studies of mammalian evolution might have been misled, particularly if there was a systematic change in the life history of the clade. However, future methods could exploit results presented here, incorporating measurements of body size as *a priori *information about the substitution rates to be inferred.

While both mitochondrial and nuclear rates show an inverse correlation with body mass, the results differ in important respects, implying that the causal mechanisms are quite different in the two sets of loci. The success of maximum lifespan as a predictor of mitochondrial rates, and its comparative failure to predict nuclear rates, provides support for theories linking mtDNA damage to aging. Causal interpretation of the nuclear results is more difficult, but one conclusion is clear. Molecular change may be decoupled from phenotypic change in the sense that many changes in germline DNA may lead to vanishingly small changes in phenotype [18,28], but mammalian molecular evolution is nevertheless a part of life history evolution, and the treatment of germline mutation rates as a component of life history deserves further attention.

## Methods

### Genetic data

All genetic sequence data were obtained from GenBank [81], and aligned by eye using Se-Al [82]. Accession Numbers are listed in the Data Supplement [Additional file 1], and alignments are available on request.

For the mitochondrial dataset, the 9 longest protein-coding genes (ATP6, COI, COII, COIII, CYTB, ND1, ND2, ND4, ND5) were aligned for 160 mammalian species. (The four short genes, less comprehensively sampled, were excluded to increase taxonomic coverage.) The resulting data set allowed us to choose at least one comparison pair from the monotremes, from 5 of the 7 marsupial orders, and from 13 of the 19 'molecular consensus' placental orders [21]. The absent orders were small, containing just 100 species in total, of which half are Afrosoricida.

For the nuclear data set, there was a clear tradeoff between alignment length and taxonomic coverage. We chose a data set consisting of partial coding sequences from 6 nuclear loci, which were available for 58 species. This data set allowed us to choose pairs only for the Eutheria, but 16 of their 19 orders were represented. Genes and approximate sequence lengths are ADRB2 ~830 bp; ATP7A ~680 bp; BDNF ~590 bp; CNR1 ~1000 bp; EDG1 ~980 bp; RAG2 ~740 bp. In addition to annotated sequences, BLAST searches were carried out on genomic contigs from *Echinops telfairi *(lesser hedgehog tenrec) and *Myotis lucifugus *(little brown bat; Lindblad-Toh, K., J. L. Chang, S. Gnerre, M. Clamp, and E. S. Lander, unpublished), confirming orthology by reciprocal BLAST.

### Life history data

In most cases, the life history data were taken from the PanTHERIA database of mammalian life history and ecology (K. E. Jones, J. Bielby, A. Purvis, D. Orme, A. Teacher, J. L. Gittleman, R. Grenyer, et al. unpublished; [22]). Body mass measurements were the unique median of adult (or age unspecified) mass, of males and females, based on the GLM equation ln(body mass) = species + sex. For two Chiropterans in the mitochondrial data set (*Rhinolophus monoceros *and *Chalinolobus tuberculatus*) this value was extrapolated from head-body length. Our measure of fecundity was the product of litter size, and litters per year. Litter size is the full median of offspring number born per litter per female, counted before birth, at birth, or after birth, based on the GLM equation ln(litter size) = species + litter size definition. Litters per year is the full median of the number of litters per female per year for non-captive individuals. Maximum lifespan was simply the maximum recorded adult age from a wild or captive individual. To supplement the PanTHERIA data, we obtained additional measurements from the database AnAge (Build 9, Feb. 2006; [83]), which contains values from multiple recent compilations from the literature. Our preferred proxy of generation time was the unique median of age at first birth. Values from the PanTHERIA database were supplemented by equivalent data from the compilation of Wooton [84]. Because coverage remained insufficient for a robust analysis, we also included some measurements where generation time was defined as age at sexual maturity plus gestation period [14], with values obtained from the AnAge database (Build 9, Feb. 2006; [83]).

### Choice of phylogenetically independent comparison pairs

For the comparative analysis, phylogenetically-independent sister pairs were chosen from the recent species-level supertree of all mammals [21,26]. Comparisons between reconstructed states at internodes [33] were not included, as these are problematic in the comparative study of substitution rates [see Additional file 2]. For three comparisons, no suitable outgroup was available, and so molecular branch lengths were estimated from a split along one of the lineages, rather than from the pair's common ancestor, making the pair paraphyletic with respect to another pair. In each case the distance of the chosen node from the common ancestor of the pair was small compared to the total divergence between the pair. Nevertheless, in all three cases, rate estimates were corrected to take into account the different time periods represented by the two molecular branch lengths. Repeating analyses with these pairs excluded was found to make little difference to the results (not shown).

### Branch length estimation

Nonsynonymous and synonymous molecular branch lengths for each pair were estimated via maximum likelihood, using the codon-based substitution model of Goldman and Yang [85,86]. Both the overall substitution rate and the ratio of nonsynonymous to synonymous changes were allowed to take branch-specific values, and so estimating branch lengths for the complete tree risks overparameterisation. Furthermore, whole-tree estimation can be unreliable if nuisance parameters, such as base composition, vary across groups. For these reasons, the data were split into small groups of 3–8 related species, and branch lengths estimated separately for these small subtrees. For both nuclear and mitochondrial data sets, results reported here are for concatenated multi-locus alignments. Analyses were also carried out for individual loci, and for a lineage-effect component of rate variation, estimated via the method of Smith and Eyre-Walker [2,4], but as results did not differ qualitatively from those obtained by the simpler method of concatenating the sequences, they are not reported further. In a few cases, one or more branches of a subtree showed signs of saturation, but the relevant pairs fit the same trends as the remainder of the data set, implying that signal was present in the sequence data, and so they were not excluded from the analyses.

Full details of the comparison pairs, the molecular branch lengths, and life history measurements are included in the Data Supplement [Additional file 1].

### Statistical tests

The independent comparisons were analysed with multiple regression, forcing all regression lines through the origin [34,35]. For results to be valid, it is important to meet the assumptions of the parametric test, and this typically involves transforming the trait measurements, and standardising comparisons to account for their different periods of divergence [33]. For this purpose, estimated divergence dates were taken from [26]. (An advantage of the use of sister pairs is that the dates were required solely for this purpose, and have no effect on the estimated differences in log rates.) To assess the standardisations and transformations used, we employed standard regression diagnostics, and customised tests [35,36]. These methods were extended to solve the special problems posed by substitution rate data [37]. The extended methods use patterns in the contrast variance to define a minimum depth below which comparison pairs were excluded [see Additional file 2]. In this way, we hoped to prevent the strength of any effect being masked by stochastic noise in the substitution process [1,4,18]. All statistical tests were implemented in R [87].

We also produced raw cross-species plots, for which the absolute rates along each lineage were calculated using the estimated divergence dates. These plots were solely illustrative, but they allowed us to include taxa excluded from the independent contrasts analysis, either because of missing life-history data for one member of the pair, or because of the diagnostic tests.

## Authors' contributions

LB and JW designed research project. JW and OBE compiled data. JW performed analyses. All authors wrote and approved the final manuscript.

## Supplementary Material

Additional file 1 Data Supplement. Spreadsheet of GenBank accession numbers, life history data, and details of phylogenetically independent species pairs. Click here for file

Additional file 2 Technical Methods Appendix. Details and justification of the comparative methods used. Click here for file
