# Supplementary material for: Correlates of substitution rate variation in mammalian protein-coding sequences
Source: BMC Evol Biol. 2008 Feb 19;8:53. doi: 10.1186/1471-2148-8-53 (PMC2289806; doi:10.1186/1471-2148-8-53)
Supplement: Additional file 2 — Technical Methods Appendix. Details and justification of the comparative methods used. [file 1471-2148-8-53-S2.pdf]

Additional File 2: Technical Methods Appendix

The parametric regression test assumes that data points are statistically independent and are associated with equal variance. To meet these demands, it is usually necessary to transform the trait measurements, and to weight contrasts to account for their different rates and periods of evolutionary change. The default options, based on a geometric Brownian Motion model of trait evolution, are to log transform the trait values, and standardise each with the square root of the total divergence time (Felsenstein 1985). As such, for body mass, the contrast variable is $\left(\ln M_1 - \ln M_2\right)/\sqrt{2t}$, where M_1 and M_2 are the body mass measurements for the two members of the species pair, and t is the estimated age of their most recent common ancestor. To assess whether these procedures succeeded in generating a set of homogeneous contrasts, tests introduced by Garland et al. (1992), and Freckleton (2000) were used. Specifically, we carried out product-moment correlation tests of the contrast magnitude, $\left|\ln M_1 - \ln M_2\right|/\sqrt{2t}$, with the standardisation factor, $\sqrt{2t}$, and with the mean transformed trait value for the pair, $\left(\ln M_1 + \ln M_2\right)/2$. If the procedures have been successful, neither correlation should differ significantly from zero.

Table S1 contains results from these tests for the mitochondrial data set, and Figure S1 shows the associated scatterplots for the body mass measurements. Fig. S1a, shows that the log transformation successfully removes any strong influence of body mass on the magnitude of the change in body mass, confirmed in Table S1 by a non-significant correlation (Freckleton 2000). Figure S1b shows that deeper pairs do tend to be associated with greater amounts of body mass evolution, reflected in a significant positive correlation between $\sqrt{2t}$ and unstandardised contrast magnitude, $\left|\ln M_1 - \ln M_2\right|$. But this inhomogeneity is removed by correcting contrasts with $\sqrt{2t}$. As Table S1 shows, the same procedures were successful for the other predictor variables, with the exception of fecundity, where a correction with $2t$, rather than $\sqrt{2t}$, was found to be necessary. (The youngest pair, *Canis lupus*-*C. latrans*, also appears to be an outlier for all traits. This may reflect an increase in evolutionary rate in these lineages, or an incorrectly

estimated date for their split; in any case this pair was excluded from the main analyses for other reasons discussed below.)

For the substitution rate contrasts, the same procedures were used, and so the contrast in synonymous substitution rates was calculated as $\left(\ln dS_1 - \ln dS_2\right)/\sqrt{2t}$. Because the species form a sister pair, substitutions will have accrued for the same period of time along both lineages, and so the difference in log branch lengths, $\ln dS_1 - \ln dS_2 = \ln\left(dS_1/dS_2\right)$, is also the maximum likelihood estimate of the difference in log substitution rates, as required. However, the time-dependent nature of the substitution process complicates tests for homogeneity of variance. The reason is that a substitution rate contrast might be associated with high variance for two quite different reasons: (1) the tendency of deeper contrasts to be associated with greater amounts of rate evolution, or (2) the fact that shallower contrasts, or slower lineages, will accumulate fewer substitutions, such that the rate estimate is associated with more stochastic noise. A successful correction of trend (1) may result in significant negative trends due to (2). Indeed, the tests of Freckleton (2000) and Garland et al. (1992) both show significant negative trends for the standardised substitution rate contrasts (Table S1 "*dS* all pairs"). These trends could be removed by leaving contrasts unstandardised (Table S1), but Welch and Waxman (in press) show that including the shallow, or slow contrasts (for which rates are the most inaccurately measured), can obscure the true relationship between rate and the predictor variable, and inflate Type II error rates. As such, scatterplots equivalent to Figure S1 were used to identify minimum ages and rates, below which stochastic fluctuations in substitution number appeared to be an important determinant of contrast magnitude. The plots for mitochondrial synonymous rates are shown in Figure S2. We chose to exclude the two slowest contrasts (identified by asterisks), and the nine shallowest contrasts (identified by empty circles). The reduced set of 45 contrasts (filled circles) gave no indications of inhomogeneity of variance. Furthermore, the reduced set of unstandardised contrasts showed a significant positive trend (Fig. S2b; Table S1) suggesting that the lack of a trend evident in the complete set of unstandardised contrasts reflects not homogeneity, but a U-shaped function, which is consistent with theoretical predictions (Welch and Waxman, in press).

Table S2 contains equivalent results for the nuclear dataset, where the same methods were used. Figure S3 shows the scatterplots for the nuclear *dS* data. Again, standardised contrasts showed a pattern of decreasing magnitude over time, suggesting that stochastic fluctuations in substitution counts are a major determinant of contrast magnitude for the shallower pairs. Again, too, we chose to exclude the nine shallowest contrasts, but here, the data from the excluded pairs were used to define three new deeper contrasts, shown as triangles in the plots [see Additional file 1].

To demonstrate the importance of the procedures set out here, consider the regression of synonymous rate on body mass for the nuclear data set. From Table 1 in the main text, this regression is highly significant when the set of deeper contrasts are used (i.e., the filled circles and triangles in Fig. S3c), but when the complete set of 22 unstandardised contrasts are used (i.e., the filled and empty circles in Fig. S3b), the relationship between rate and trait is obscured ($n = 22$; $r^2 = 0.054$; $p = 0.287$), presumably due to the inaccuracy of the rate estimates obtained from the shorter branches.

In addition to the tests described above, which are specifically designed for phylogenetic comparative methods, all regressions were subjected to the suite of diagnostic tests implemented in R (R Development Core Team 2006), and the Shapiro-Wilks test of normality.

In common with most comparative studies of substitution rates, we chose not to increase our sample size by calculating additional contrasts between reconstructed states at the internal nodes of the phylogeny (Felsenstein 1985). In addition to the possibility of overparameterisation in the rate estimates, such contrasts are problematic for a number of reasons (Welch and Waxman, in press). First, for traits such as body size, the weighted averages used to generate internodal contrasts need not represent reconstructions of ancestral states, and analyses including only such traits can succeed even if these weighted averages deviate substantially and systematically from the true ancestral states (Grafen 1989). This is not true, however, for rate contrasts, because internal molecular

branch lengths are unavoidably reconstructions of ancestral states, and so, unlike the weighted averages, should reflect evolutionary changes in the early history of the clade. Second, because our date estimates for the internal nodes came themselves from molecular data (Bininda-Emonds et al. 2007), serious errors of sign (i.e., the misidentification of the faster lineage) can arise in the calculation of internodal contrasts, but not for contemporaneous sister pairs, where the two lineages are the same age by definition. Finally, analyses using internodal contrasts can be seriously misled by the node density artefact (see, e.g., Davies and Savolainen 2006).

References

Bininda-Emonds, O. R. P., M. Cardillo, K. E. Jones, R. D. E. MacPhee, R. M. D. Beck, R. Greyner, S. A. Price, R. A. Vos, J. A. Gittleman and A. Purvis. 2007. The delayed rise of the present-day mammals. Nature 446: 507-512.

Davies, T. J., and V. Savolainen. 2006. Neutral theory, phylogenies, and the relationship between phenotypic change and evolutionary rates. Evolution 60: 476-483.

Felsenstein, J. 1985. Phylogenies and the comparative method. Am. Nat. 125: 1-15.

Freckleton, R. P. 2000. Phylogenetic tests of ecological and evolutionary hypotheses: checking for phylogenetic independence. Functional Ecology 14: 129-134.

Garland, T. Jr, P. H. Harvey, and A. R. Ives. 1992. Procedures for the analysis of comparative data using phylogenetically independent contrasts. Syst. Biol. 41: 18-32.

Grafen, A. 1989. The phylogenetic regression. Philos. T. R. Soc. B. 326: 119-157.

R Development Core Team. 2006. R: a language and environment for statistical computing. V 2.4 See http://www.R-project.org.

Welch, J. J. and D. Waxman. Calculating independent contrasts for the comparative study of substitution rates. J. Theor. Biol. In press.

Table S1: Correlation tests of homogeneity of variance for mitochondrial data set

		(*a*)		(*b*)		(*c*)	
Trait	*n*	*r*	*p*-value	*r*	*p*-value	*r*	*p*-value
Body mass	56	0.19	0.15	0.41	0.0015**	-0.14	0.30
Max. lifespan	46	0.07	0.66	0.38	0.0087*	-0.18	0.24
Generation time	37	-0.20	0.24	0.20	0.23	-0.29	0.086
Fecundity †	36	0.19	0.26	0.58	0.00020**	-0.29	0.081
dS (all pairs)	56	-0.29	0.028*	-0.01	0.94	-0.43	0.0011**
dS (older/faster pairs)	45	-0.17	0.27	0.39	0.0086*	-0.04	0.80
dN (all pairs)	56	-0.07	0.61	-0.00	0.97	-0.41	0.0017**
dN (older/faster pairs)	45	-0.05	0.76	0.08	0.59	-0.21	0.16

Note: (a) standardised contrast magnitude and mean transformed trait value; (b) unstandardised contrast magnitude and the contrast standard deviation; (c) standardised contrast magnitude and the contrast standard deviation; * $p<0.05$; ** p<0.005. † contrasts standardised with $2t$ rather than $\sqrt{2t}$.

Table S2: Correlation tests of homogeneity of variance for nuclear data set

		(*a*)		(*b*)		(*c*)	
Trait	*n*	*r*	*p*-value	*r*	*p*-value	*r*	*p*-value
Body mass	22	0.30	0.18	0.20	0.36	-0.27	0.23
Max. lifespan	22	-0.26	0.25	0.50	0.018*	0.23	0.31
Generation time	18	-0.10	0.69	0.25	0.32	-0.11	0.66
Fecundity †	15	0.17	0.53	0.08	0.78	-0.51	0.056
dS (all pairs)	22	-0.39	0.074	-0.13	0.57	-0.58	0.0050**
dS (older pairs)	16	0.45	0.084	0.44	0.092	0.31	0.25
dN (all pairs)	22	0.05	0.81	-0.17	0.44	-0.59	0.0041**
dN (older pairs)	16	-0.48	0.061	-0.02	0.94	-0.17	0.52

Note: (a) standardised contrast magnitude and mean transformed trait value; (b) unstandardised contrast magnitude and the contrast standard deviation; (c) standardised contrast magnitude and the contrast standard deviation; * $p<0.05$; ** p<0.005. † contrasts standardised with $2t$ rather than $\sqrt{2t}$.

Figure Captions

Figure S1:

Diagnostic plots for the body mass contrasts from the mitochondrial data set. Part (a) shows the standardised contrast magnitudes against the mean log transformed trait value for each pair. Part (b) shows the unstandardised contrast magnitudes against the standard deviation of the contrast expected under a geometric Brownian Motion model of trait evolution. Part (c) is identical to (b), but with contrasts standardised with their standard deviations. The contrast value is notated as $\Delta \ln M = \ln M_1 - \ln M_2$, where M_1 and M_2 are the body mass measurements in grammes for the two members of the pair, and t denotes the estimated age of the pair's most recent common ancestor in million years before present.

Figure S2:

Diagnostic plots for the mitochondrial synonymous rate contrasts. In these plots, $r = dS/t$, denotes the estimated substitution rate per site, with all other details as in Figure S1. Contrasts were excluded from the analysis if trends in the contrast magnitudes indicated that the change in rate could not be reliably estimated from the molecular branch lengths. The excluded points are indicated with empty circles (contrasts deemed too shallow), or asterisks (contrasts where rates were too slow).

Figure S3:

Diagnostic plots for the nuclear synonymous rate contrasts. All details are as for Figure S2 with the addition that triangles indicate the three new deeper contrasts, constructed from lineages excluded from the analysis (empty circles).

Figure S1

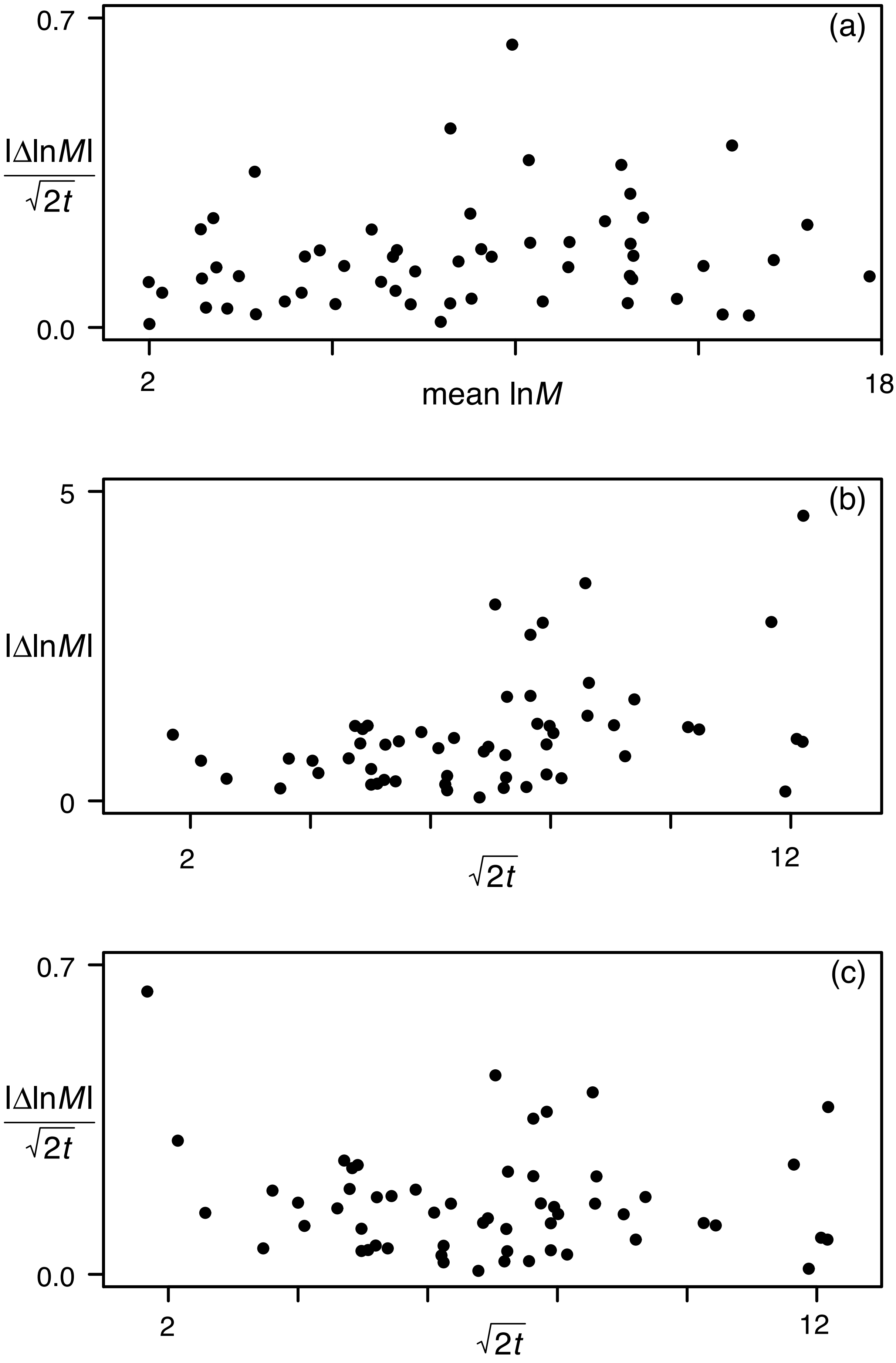

Figure S2

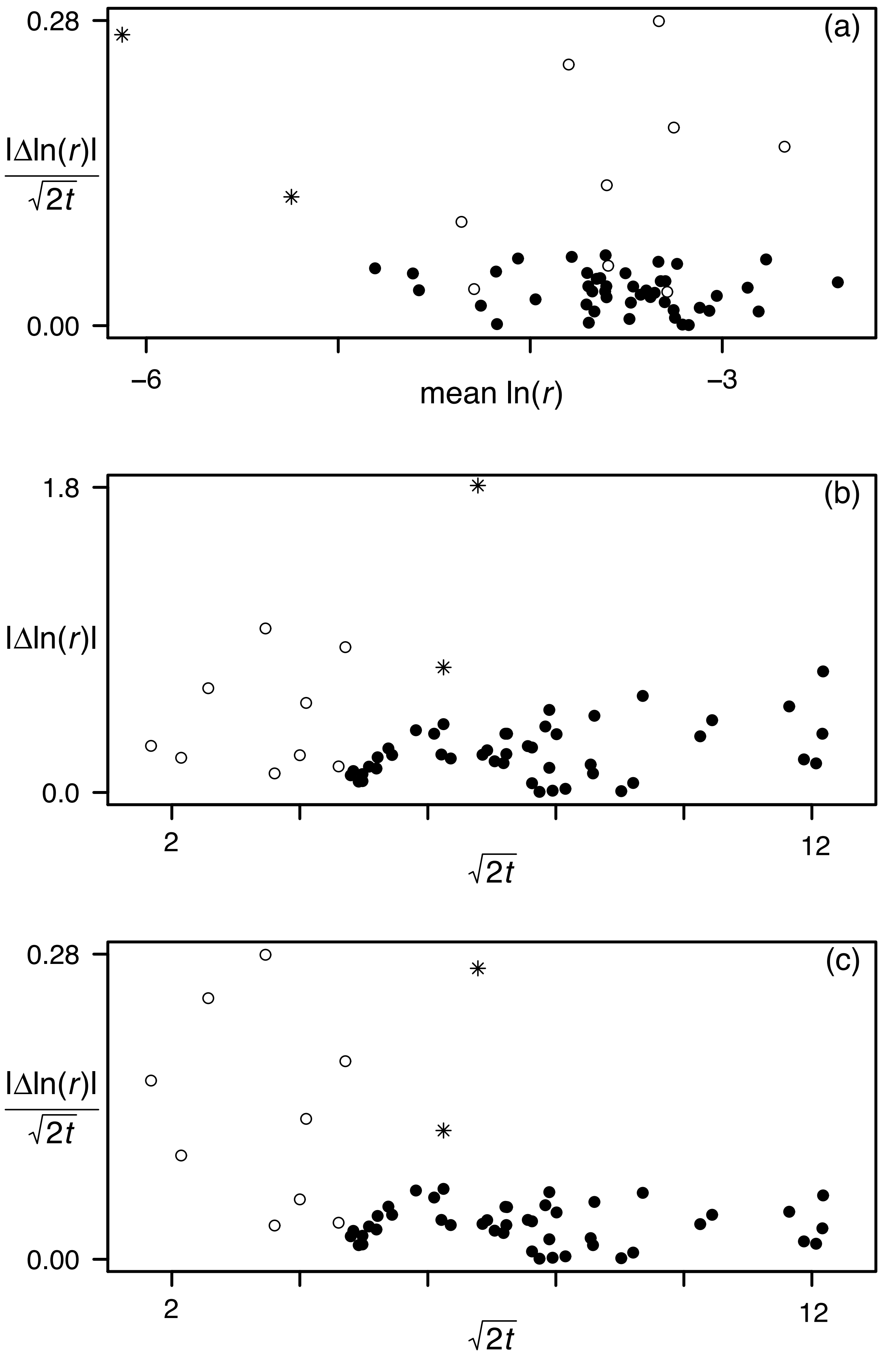

0.28
0.00
(a)
|Δln(r)|
√2t
−6
−3
mean ln(r)
1.8
0.0
(b)
|Δln(r)|
2
12
√2t
0.28
0.00
(c)
|Δln(r)|
√2t
2
12
√2t

Figure S3

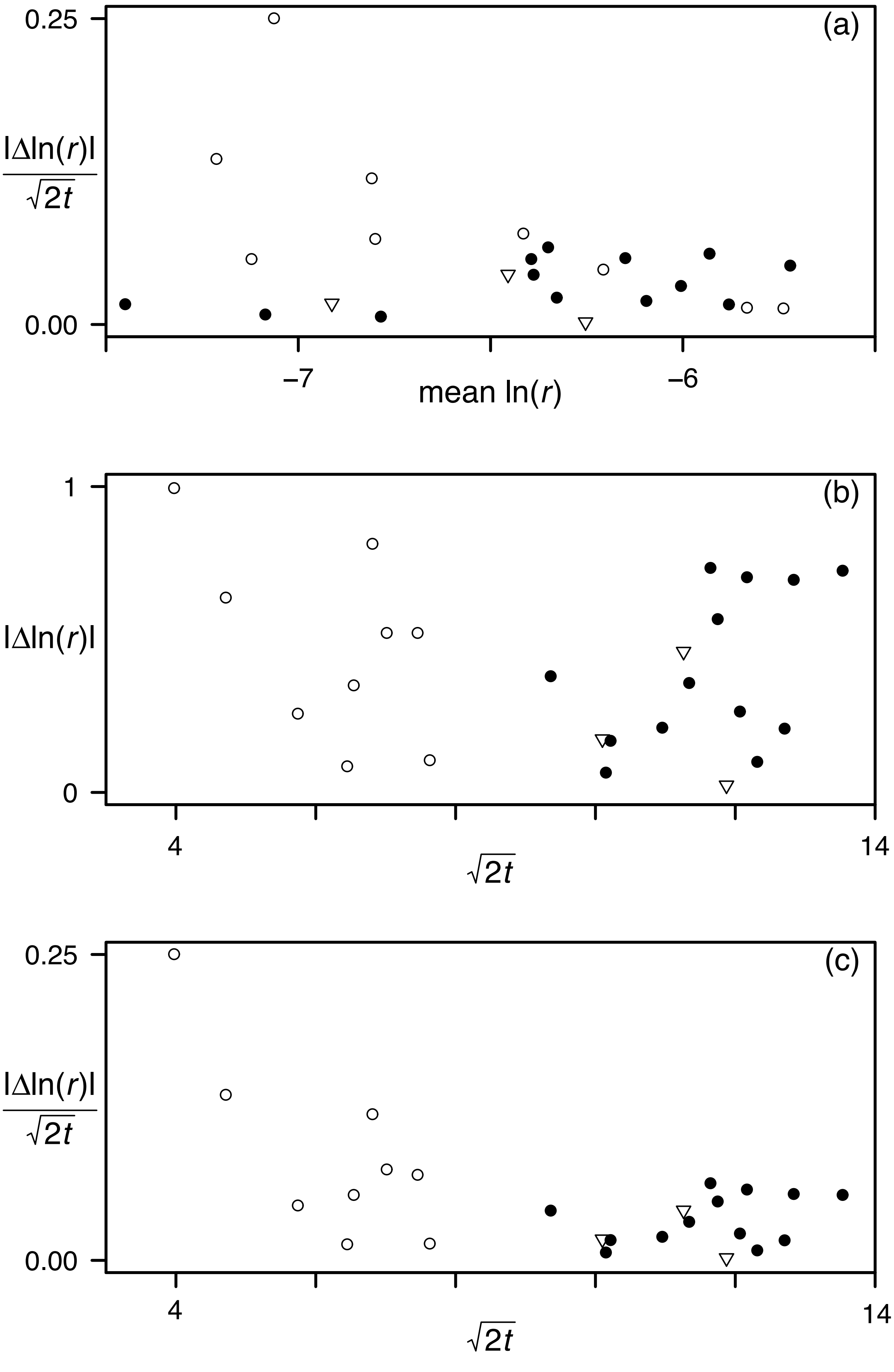
(a)
0.25
0.00
$\frac{|\Delta \ln(r)|}{\sqrt{2t}}$
−7
−6
mean ln(r)
(b)
1
0
|Δln(r)|
4
14
$\sqrt{2t}$
(c)
0.25
0.00
$\frac{|\Delta \ln(r)|}{\sqrt{2t}}$
4
14
$\sqrt{2t}$
